# Zinc Deficiency in Critically Ill Patients: Impact on Clinical Outcome

**DOI:** 10.7759/cureus.61690

**Published:** 2024-06-04

**Authors:** Pradeep K Suruli, Pradeep Rangappa, Ipe Jacob, Karthik Rao, Sweta Shivashanker

**Affiliations:** 1 Department of Critical Care Medicine, Manipal Hospital Yeshwantpur, Bengaluru, IND; 2 Department of Biochemistry, Manipal Hospital Yeshwantpur, Bengaluru, IND

**Keywords:** severity of illness, zinc sequestration, zinc deficiency, stress response, oxidative stress, nutritional immunity, inflammatory biomarkers, hypozincemia, acute phase response

## Abstract

Background

Zinc is a trace element essential for the normal functioning of many vital enzymes and organ systems. Studies examining the rates and degrees of zinc deficiency and its consequences in patients with critical illnesses remain scarce.

Materials and methods

This is a prospective observational study assessing zinc deficiency in critically ill adult patients admitted to a tertiary care intensive care unit (ICU) and its impact on clinical outcomes. Patients were divided into those with normal (≥ 71 µg/dl) and low (≤ 70 µg/dl) zinc levels. Zinc-deficient patients were further divided into mild, moderate, and severe zinc deficiency groups based on zinc levels of 61-70 µg/dl, 51-60 µg/dl, and below 51 µg/dl, respectively. The primary outcome assessed was ICU mortality, and the secondary outcomes were ICU length of stay (LOS), duration of invasive mechanical ventilation (IMV), acute kidney injury (AKI) at admission, need for non-invasive ventilation (NIV), renal replacement therapy (RRT), or vasopressors during the course of the ICU. Other parameters compared included APACHE (Acute Physiology and Chronic Health Evaluation) II, SOFA (Sequential Organ Failure Assessment) score on day 1, and levels of lactate, procalcitonin, calcium, magnesium, phosphate, and serum albumin. The study also compared the mean zinc levels in patients with low and high SOFA scores (scores up to 7 vs. 8 and above) and low and high APACHE II values (scores up to 15 vs. 16 and above).

Results

A total of 50 patients were included, of whom 43 (86%) were zinc deficient. Mortality in zinc-deficient and normal zinc-level patients was 33% and 43%, respectively (p = 0.602). Patients with zinc deficiency were also older (mean age 69 vs. 49 years, p = 0.02). There was no difference in secondary outcome parameters, except for more zinc-deficient patients needing RRT. Twenty-six of the zinc-deficient patients had severe zinc deficiency, ten moderate, and seven mild (p = 0.663). ICU mortality was approximately 42%, 10%, and 29% in the severe, moderate, and mild deficiency groups, respectively (p = 0.092). Zinc levels were similar between those with low and high APACHE II scores (mean 47.9 vs. 45.5 µg/dl, p = 0.606) as well as between low and high SOFA scores (mean 47.8 vs. 45.7 µg/dl, p = 0.054).

Conclusion

The present study suggests that zinc deficiency is very common in critically ill patients but does not correlate with their severity of illness, nor does it lead to a poorer outcome in these patients. However, further studies with a larger cohort of patients would be required to make definitive conclusions.

## Introduction

Zinc (Zn) is the second-most abundant intracellular trace element after iron [1]. It is a very versatile entity and is required for the normal functioning of the immune system, oxidative stress responses, glucose control, neurocognitive function, and wound healing [2]. It plays a structural role in proteins, a catalytic role in six enzyme classes, namely dehydrogenases, transferases, hydrolases, lyases, isomerases, and ligases, and a regulatory role in endocrine, paracrine, and autocrine systems [3-5]. The copper/zinc-superoxide dismutase (Cu/Zn-SOD) enzyme converts superoxide into the less harmful hydrogen peroxide and oxygen; hence, zinc is essential in the defense against the increased oxidative stress seen in critical illness [6,7]. Zinc is also anti-apoptotic, acts as a co-factor in DNA synthesis, and plays a crucial role in the development and regulation of immune cells such as monocytes, neutrophils, T and B lymphocytes, dendritic cells, and natural killer (NK) cells [8]. Zinc also acts as a membrane stabilizer and maintains the integrity of the intestinal epithelial barrier [3].

Due to the ubiquitous role of zinc in multiple systems and processes, its deficiency has been shown to have far-reaching consequences. The production of pro-inflammatory cytokines such as interleukins (IL)-10, -6, -8, and -1β, interferon-c, monocyte chemoattractant protein-1, macrophage inflammatory protein-1 α, and tumor necrosis factor-α increases with decreasing serum zinc levels, especially in sepsis due to upregulation by pathogens and also in inflammatory conditions [9,10].

In the intensive care unit (ICU), lower serum zinc levels have been associated with a higher risk of recurrent sepsis [11], acute respiratory distress syndrome, ventilator-induced lung injury, and ventilator-associated pneumonia [8,12,13]. Zinc has significant anti-viral properties that differ from virus to virus, such as inhibition of viral polymerase and polyprotein cleavage in rhinoviruses, DNA polymerase in the herpes simplex virus, reduction of respiratory syncytial virus titer, and the zinc-finger anti-viral protein (ZAP), which attenuates viral protein expression in influenza. This may account for the more severe pneumonia seen in zinc-deficient patients [13]. Zinc deficiency may also worsen heart failure through stress-induced damage to cardiomyocytes and myocardial damage from cytokines [6]. Lower zinc levels have also been associated with higher Child-Pugh and Model for End-stage Liver Disease scores in chronic liver disease (CLD) due to impairment of the urea cycle enzyme, ornithine transcarbamylase in the liver, and glutamine synthetase in muscle, leading to hyperammonemia seen in hepatic encephalopathy [1,14].

Zinc present in plasma accounts for just 1% of the total body zinc content [15,16]. Almost 95% of body zinc is located intracellularly, with about 50% in skeletal muscle, 37% in bone, 4% in the liver, 4% in skin, and the remaining 1-2% in hair and other organs. About 70% of plasma zinc is bound to albumin and forms >90% of the exchangeable zinc pool. Another 10-20% is firmly bound to α-2-macroglobulin and is non-exchangeable. As such, even a small part of the plasma zinc pool entering the tissues would lead to a sharp drop in serum zinc levels [17]. For instance, 1% of the zinc pool entering the liver would lead to a 40% decrease in serum zinc.

Normal serum zinc level is 70-120 µg/dL; values below which are defined as zinc deficiency [18]. Plasma and serum zinc levels are similar, and these terms are interchangeable. Oxidative damage in critical illness leads to the impairment of the strong homeostatic mechanisms in place to prevent deviations in serum zinc when dietary intakes fluctuate, leading to decreased absorption from the gastrointestinal tract, increased urinary losses, and decreased levels of transport proteins such as albumin [10,19-21]. Increased levels of inflammatory cytokines induce the production of Zrt-Irt-like proteins (ZIP) in the liver, mainly ZIP-14, which transport zinc into the hepatocytes. There is also increased production of the intracellular metal-binding protein metallothionein. This leads to the sequestration of zinc in the liver and other organs during the stress response, leading to hypozincemia in 95% of ICU patients. This response is part of nutritional immunity, which is a host defense mechanism to limit the availability of essential micronutrients to pathogens [11]. The serum zinc pool circulates about five times daily to replenish zinc levels. However, if zinc intake is low, this pool is unable to compensate, and serum concentrations will decline within several days [17]. Other factors contributing to zinc deficiency in the initial days of ICU admission include increased metabolic rate, inadequate nutritional intake, and ongoing feeding difficulties [22].

The present study was conducted to evaluate the rates of zinc deficiency in patients admitted to a tertiary care ICU and its impact on ICU outcomes.

## Materials and methods

Study design and aim

This was a prospective observational study of zinc deficiency in adult patients admitted to a tertiary care ICU over a one-month period in November 2023. The study aimed to evaluate the rates and degrees of zinc deficiency and its effect on clinical outcomes in critically ill patients. The study also evaluated the corresponding levels of albumin, which is needed for zinc homeostasis, along with three other essential elements, calcium, phosphate, and magnesium, as well as the biomarkers of illness, lactate and procalcitonin.

Outcome measures

The primary outcome measure evaluated was ICU mortality. Secondary outcome measures included ICU length of stay (LOS), hospital LOS, duration of invasive mechanical ventilation (IMV), number of patients needing non-invasive ventilation (NIV), renal replacement therapy (RRT) or vasopressors, and the number of patients developing acute kidney injury (AKI). In addition to serum zinc, serum levels of albumin, calcium, phosphate, magnesium, lactate, and procalcitonin were also evaluated.

Study population

The study included all adult patients admitted to the ICU over a one-month period. Patients with CLD and other liver dysfunctions were excluded. Patients were divided into two groups: those with normal zinc levels (≥71 µg/dl) and those with zinc deficiency (≤70 µg/dl). Zinc-deficient patients were further classified into three groups: severe deficiency (≤50 µg/dl), moderate deficiency (51-60 µg/dl), and mild deficiency (61-70 µg/dl) [3,6].

Ethical considerations

The study protocol was reviewed and approved by the Institutional Review Board (IRB). The IRB deemed patient consent unnecessary because there was no direct intervention involving the patients.

Data collection and statistical analysis

Data were collected from patient charts and electronic medical records. Population data were expressed in terms of numbers and percentages. Other data, including LOS, SOFA, and APACHE II scores and serum levels, were expressed in terms of median and inter-quartile range (IQR), and the serum levels of zinc and other elements in terms of mean and standard deviation (SD). They were analyzed using IBM SPSS Predictive Analytics Software Statistics, Version 20 (IBM Corp., Armonk, NY, USA). Comparisons between groups according to serum zinc level were tested for significance by the Student's t-test and Pearson chi-square tests. P < 0.05 was considered statistically significant.

## Results

Out of a total of 50 people included in the study, 43 (86%) were zinc deficient (p = 0.002) (Table 1). They were older than those who had normal zinc levels (mean age: 68.8 vs. 48.9 years, p = 0.02). ICU mortality did not differ between the two groups (32.5 vs. 42.8%, p = 0.642); there was also no difference in gender, secondary outcome parameters, severity of illness, levels of lactate and procalcitonin, and levels of calcium, phosphorus, and magnesium between either group. Although zinc deficiency patients did not have a higher prevalence of AKI, their need for RRT was higher (11.6 vs. 28.6%, p = 0.02).

**Table 1:** Comparison of outcome and other laboratory values in zinc deficiency versus normal zinc ICU: intensive care unit, IQR: interquartile range; LOS: length of stay, IMV: invasive mechanical ventilation, NIV: non-invasive ventilation, SOFA: Sequential Organ Failure Assessment, APACHE II: Acute Physiology and Chronic Health Evaluation II, RRT: renal replacement therapy

		Low zinc (n = 43) median (IQR)	Normal zinc (n = 7) median (IQR)	p-value
No. of patients		43/50 (86%)	7 (14%)	0.002
Age (mean, SD)		68.8 ± 13.38	48.9 ± 18	0.02
Gender	Male	27/43 (63%)	5/7 (71%)	0.675
	Female	16/43 (37%)	2/7 (29%)	
ICU mortality		14/43 (32.5%)	3/7 (42.8%)	0.642
ICU LOS		7 (5–11)	6 (5–11)	0.60
Hospital LOS (days)		11 (7–14)	8 (6–14)	0.961
Duration of IMV (days)		0 (0–7)	0 (0–0)	0.058
SOFA (day 1)		6 (4–10)	8 (3–9)	0.496
APACHE II		14 (9–22)	18 (9–24)	0.886
Patients needing NIV		14/43 (32.5%)	2/7 (28.5%)	0.846
Patients with AKI		21/43 (48.8%)	4/7 (57.1%)	0.711
Patients needing RRT		5/43 (11.6%)	2/7 (28.6%)	0.02
Patients needing vasopressor		17/43 (39.5%)	2/7 (28.6%)	0.597
Serum albumin		2.7 (2.2–3)	2.5 (2–3)	0.767
Serum lactate		2 (1.5–3.2)	2.5 (1–6)	0.477
Serum procalcitonin		0.78 (0.5–2.4)	1 (0.78–2)	0.113
Serum calcium		7.6 (7–8)	8 (7–9)	0.633
Serum phosphate		2.9 (2.7–4)	3.7 (3–3.8)	0.529
Serum magnesium		1.9 (1.7–2)	1.8 (1.5–2)	0.482

Of the 43 patients in the zinc deficiency group, 26 (60%) were severely deficient (serum zinc ≤50 µg/dl), 10 (23%) were moderately deficient (serum zinc 51-60 µg/dl), and 7 (16%) were mildly deficient (serum zinc 61-70 µg/dl) (Table 2). There was no difference in demographic data, primary or secondary outcome parameters, the severity of illness, biomarkers, or levels of calcium, magnesium, or phosphorus between the three groups.

**Table 2:** Comparison of outcome parameters among mild, moderate and severe zinc deficiency patients IQR: interquartile range; ICU: intensive care unit, LOS: length of stay, IMV: invasive mechanical ventilation, NIV: non-invasive ventilation; SOFA: Sequential Organ Failure Assessment; APACHE II: Acute Physiology and Chronic Health Evaluation II; RRT: renal replacement therapy

Variable		Degrees of zinc deficiency			p-value
		Severe (serum Zn ≤ 50 µg/dl) median [IQR]	Moderate (serum Zn 51–60 µg/dl) median [IQR]	Mild (serum Zn 61–70 µg/dl) median [IQR]	
No. of patients		26 (60%)	10 (23%)	7 (16%)	
Age (mean ±SD)		68.4 ± 12.7	71.8 ± 7.6	65.9 ± 13.4	0.659
Gender	Male	17/26 (65.4%)	6/10 (60%)	4/7 (57.1%)	0.663
	Female	9/26 (34.6%)	4/10 (40%)	3/7 (42.9%)	
ICU mortality		11/26 (42.3%)	1/10 (10%)	2/7 (28.6%)	0.092
ICU LOS (days)		7.0 [5.0–11.0]	8.5 [4.0–18.5]	7.0 [5.0–9.0]	0.791
Hospital LOS (days)		9.5 [7.0–13.8]	5.3 [11.5–20.8]	12.0 [7.0–14.0]	0.754
Duration of IMV (days)		1.0 [0.0–7.8]	0.0 [0.0–15.8]	0.00 [0.0–5.0]	0.733
SOFA (day 1)		7.0 [4.0–11.8]	5.5 [3.3–8.3]	7.0 [3.0–13.0]	0.636
APACHE II		14.0 [10.5–25.3]	11.5 [7.5–19.3]	13.0 [8.0–28.0]	0.407
Patients needing NIV		7/26 (26.9%)	4/10 (40%)	2/7 (28.6%)	0.559
Patients having AKI		13/26 (50.0%)	5/10 (50.0%)	3/7 (42.9%)	0.850
Patients needing RRT		4/26 (15.4%)	1/10 (10%)	0/7 (0.0%)	0.342
Patients needing vasopressors		12/26 (46.1%)	1/10 (10%)	4/7 (57.1%)	0.780
Serum albumin		2.5 [2.0–3.2]	2.9 [2.6–3.0]	2.5 [2.4–3.0]	0.926
Serum calcium		7.5 [6.8–8.0]	7.9 [7.2–8.2]	7.4 [6.8–8.2]	0.232
Serum phosphate		3.0 [2.7–4.0]	2.8 [2.2–3.0]	3.0 [1.7–4.1]	0.239
Serum magnesium		1.9 [1.8–2.1]	2.0 [1.6–2.2]	1.9 [1.5–2.0]	0.890
Lactate		2.0 [1.6–3.2]	1.9 [1.1–2.5]	2.0 [1.1–4.0]	0.676
Procalcitonin		1.5 [0.5–3.0]	0.5 [0.1–0.6]	0.7 [0.5–2.8]	0.086

Severe zinc deficiency was seen in malignancy, septic shock, traumatic brain injury, and pneumonia (Table 3) and accounted for 52% of the patients included in the study.

**Table 3:** Admission diagnosis and serum zinc levels in zinc deficient patients

Diagnosis	No. of patients	Mean zinc level (µg/dl)
Acute coronary syndrome	1	55
Acute kidney injury	1	51
Guillain Barre syndrome	1	60
Gastrointestinal hemorrhage	1	67
Malignancy	1	33
Pneumonia	15	47.6
Polytrauma	2	58
Seizures	2	61.5
Septic shock	14	40.1
Stroke	3	50.7
Traumatic brain injury	2	40.5
Total	43	51.3

Table 4 compares zinc levels between patients with low and high severity of illness scores. There was no difference in zinc levels between patients with low and high SOFA scores on day 1 or between those with low and high APACHE II scores.

**Table 4:** Zinc levels according to severity of illness based on SOFA and APACHE II scores SOFA: Sequential Organ Failure Assessment; APACHE: Acute Physiology and Chronic Health Evaluation

Comparison of zinc levels in low and high SOFA groups			
	Group A (n = 24) SOFA score up to 7 (mean ± SD)	Group B (n = 19) SOFA score ≥ 8 (mean ± SD)	P-value
Zinc level (µg/dl)	47.8 ± 12.6	45.7 ± 12.6	0.606
Comparison of zinc levels in low and high APACHE II groups			
	Group A (n = 24) APACHE II score up to 15 (mean ± SD)	Group B (n = 19) APACHE II score ≥ 16 (mean ± SD)	P-value
Zinc level (µg/dl)	47.9 ± 12.6	45.5 ± 12.6	0.540

## Discussion

To the best of our knowledge, this is the first Indian study assessing the levels of zinc deficiency and outcomes in non-CLD patients admitted to the ICU, as well as the difference in outcome between mild, moderate, and severe zinc deficiency. Zinc deficiency is common in the developing world due to the consumption of high-phytate-containing cereal proteins and may affect nearly 2 billion people [15,16,22]. Globally, the prevalence of zinc deficiency ranges from 4% in countries with predominantly animal protein-based diets to 73% in countries with plant-based diets. The results of the present study show that 86% of critically ill patients admitted to the ICU were zinc deficient, of whom 60% had severe zinc deficiency with levels ≤50 µg/dl. Other studies in critically ill patients have shown similarly high levels of zinc deficiency, ranging from 75% to 95% [7,20,23]. Table 5 enlists the common ICU conditions associated with zinc deficiency [3,5,6,14,15,21,24-30].

**Table 5:** Common conditions in ICU associated with zinc deficiency

Anemia
Bariatric surgery involving bypass procedures
Burns cover 30% of body surfaces
Chronic conditions associated with hypoalbuminemia, such as diabetes, liver disease, sickle cell disease, kidney disease, alcoholism, and HIV
Ferrous sulfate through nasogastric feeds impairs Zn absorption
Excessive gastrointestinal losses (diarrhea, high output fistula, bile leak due to bile duct injury)
Gastrointestinal edema or impaired motility causing decreased absorption
Heart failure
High dose steroids
Malignancies
Pancreatic insufficiency
Porto-systemic shunts
Prolonged renal replacement therapy - due to loss in dialysate or adsorption by the filters
Respiratory failure
Sarcopenia
Severe trauma and traumatic brain injury
Systemic inflammatory response syndrome (serum zinc levels fall as C-reactive protein levels increase)
Small bowel malabsorption seen in short bowel syndrome, Crohn’s disease, hookworm infestation, etc.
Post-surgery (due to increased muscle catabolism after surgery)
Total parenteral nutrition – long term

Symptoms suggestive of zinc deficiency in ICU patients include unexplained hyperglycemia, muscle weakness leading to respiratory distress, worsening hepatic dysfunction, altered taste and smell, diarrhea, rash in extremities, and poor wound healing [7,21,24]. Mild forms of zinc deficiency may be difficult to diagnose due to the absence of overt clinical features, and serum levels may remain within the normal range [3,5].

Zinc deficiency was not associated with increased mortality in this study. There was also no difference in ICU or hospital LOS or in APACHE II or SOFA scores on day 1 between the two groups. There was also no difference in these parameters between patients with severe, moderate, or mild zinc deficiency. Most ICU studies evaluating the correlation of zinc deficiency with clinical outcomes and severity of illness scores have shown conflicting results [2,11,22,23,25,28,31-35]. Most of these were small studies with less than 100 patients. A systematic review by Rodic et al. showed no clear association between zinc levels and ICU or hospital length of stay [34]. An observational study by Lee et al. with 167 ICU patients showed that zinc deficiency was associated with a shorter hospital LOS but had no bearing on mortality, ICU LOS, APACHE II, or SOFA scores [30]. Ekemen Keleş et al. reported that zinc-deficient pediatric patients with sepsis had higher SOFA scores but lower 28-day mortality and no difference in APACHE II score. [31] Similarly, a randomized control trial (RCT) by Berger et al. with 200 post-surgery patients with organ failure showed no effect of zinc deficiency on the SOFA score [32]. A prospective study on 487 traumatic brain injury patients by Kim et al. showed higher odds for one-month mortality and disability in those with hypozincemia [27].

In the present study, zinc-deficient people were older than those with normal zinc levels (mean 69 vs. 49 years, respectively), similar to other studies [7,9,12,24]. Old age is a risk factor for zinc deficiency, especially in those from long-term care facilities, due to poor nutrition, poor absorption, and the presence of chronic conditions such as diabetes mellitus or obesity.

Albumin is the major zinc-binding protein in plasma and is the major determinant of plasma zinc levels [4]. Conditions leading to low serum albumin, such as inflammation, liver disease, sepsis, burns, and any cause of critical illness, are often associated with a corresponding fall in plasma zinc levels [3,11,17,21]. The rate of gastrointestinal zinc absorption is also associated with serum albumin levels. Another factor correlating albumin and zinc is the level of free fatty acids [3]. Free fatty acids bind strongly to albumin, blocking its zinc-binding site and lowering its zinc-binding capacity. Hence, conditions with increased free fatty acids, both physiological (e.g., fasting, stress, extreme exercise) and pathophysiological (obesity, diabetes, liver disease), may be associated with decreased body zinc content. In the present study, hypoalbuminemia did not appear to correlate with zinc deficiency, as both groups with low and normal zinc levels had low serum albumin. This study excluded CLD patients as there is a well-established association between CLD and zinc deficiency due to hypoalbuminemia, poor absorption from the small intestine, and increased urinary secretion due to increased binding to α2 macroglobulin [1,14,20].

This study showed severe zinc deficiency in septic shock and pneumonia patients, in keeping with the findings of other studies and the rationale for increased oxidative stress and inflammatory biomarkers in sepsis, especially lipid peroxidation, IL-6, and TNF-α, leading to increased sequestration of zinc in the liver [9,19,31]. The present study did not find any difference in the levels of the inflammatory biomarkers lactate and procalcitonin. Other studies have shown decreased levels of other essential elements in hypozincemia, such as calcium, iron, and sodium, and trace elements, such as selenium and copper [6,19,23]. However, in the present study, levels of calcium, magnesium, and phosphorus were not decreased in zinc-deficient patients.

The only significant secondary outcome in this study was the greater need for RRT in zinc-deficient patients. There was no difference in the number of patients on IMV or needing NIV or vasopressor support. The various degrees of zinc deficiency assessed in this study, mild, moderate, and severe, did not differ in terms of mortality, ICU or hospital LOS, severity of illness scores, levels of lactate, procalcitonin, albumin, calcium, magnesium, and phosphorus, and need for vasopressor support, RRT, NIV, or IMV.

The question of whether low serum zinc levels need to be corrected remains debatable. In the present study, zinc-deficient patients received a single intravenous dose of 10 mg of zinc chloride. Zinc supplementation, in many studies, has been shown to have no clear benefit [32,33] and may even be detrimental [23]. Hypozincemia induced by the acute phase response in sepsis may be protective by limiting the amount of zinc available for bacterial growth and also by limiting the cytokine storm in inflammation [2,11]. Hence, low serum Zn levels may not indicate deficiency and may simply reflect redistribution. In addition, it has been shown that serum zinc levels are unrelated to zinc supplementation, and doubling zinc intake raised serum zinc by only 6% [17]. However, studies by Xia et al. [22] and Lee et al. [30] suggest that increased serum zinc levels after correction, especially with a combination of zinc, selenium, copper, and manganese, may improve survival. The benefit of a combination of these trace elements in improving ICU outcomes has been affirmed in a recent meta-analysis [36]. In decompensated CLD, it may also prevent hepatic encephalopathy. [1]

It is also recommended to correlate zinc levels with CRP and albumin levels and to use caution when diagnosing zinc deficiency in the background of high CRP >20 mg/L, hypoalbuminemia, or anemia [5,26]. It is recommended that testing zinc levels be restricted to conditions with a risk of zinc deficiency, such as increased gastrointestinal loss as in severe diarrhea, bile loss, burns, long-term TPN or RRT, chronic pancreatic insufficiency, and short gut syndrome.

Drawbacks

The drawbacks of the present study are the small number of patients included, the fact that SOFA scores were not repeated at ICU discharge and the fact that correlative CRP levels were not measured. Although low zinc levels were corrected, follow-up serum zinc was not measured.

## Conclusions

In the present study, zinc deficiency was seen in 85% of critically ill patients admitted to the ICU. It had no impact on ICU mortality, ICU or hospital length of stay, or other ICU outcome parameters. As serum zinc levels cannot be used as a reliable prognostic marker, the question of whether there is any benefit to testing zinc levels in all ICU patients remains largely unanswered. Also, as there was no difference in outcome parameters between mild, moderate, and severe zinc deficiency patients, correcting low serum zinc levels may not be beneficial. To date, most studies evaluating zinc deficiency have shown conflicting results in terms of ICU outcomes. Further randomized control trials with a larger cohort of patients may be helpful in drawing more definitive conclusions.
